# Double encapsulation of C_60_, [6]CPP and Li^+^@C_60_ inside a peropyrene-linked, CPP-based double nanohoop†

**DOI:** 10.1039/d6qo00235h

**Published:** 2026-04-08

**Authors:** Lei Ye, Yong Yang, Michal Juríček, Thomas Drewello

**Affiliations:** a Physical Chemistry I, Department of Chemistry and Pharmacy, Friedrich-Alexander-Universität Erlangen-Nürnberg Erlangen 91058 Germany thomas.drewello@fau.de; b Department of Chemistry, University of Zurich Zurich 8057 Switzerland michal.juricek@chem.uzh.ch; c School of Chemistry and Chemical Engineering, Southeast University Nanjing Jiangsu 211189 China

## Abstract

The peculiar solid-state packing of a peropyrene-linked and CPP-based double nanohoop is known from earlier studies to hinder the desired formation of 1 : 2 complexes with C_60_. In proof-of-concept experiments presented in this work, we provide evidence of the elusive 1 : 2 complex by employing electrospray ionization mass spectrometry. Additionally, for the first time, a binary 1 : 2 ring-in-ring complex of the double nanohoop with [6]CPP is observed. Most remarkably, we succeeded in generating a stable 1 : 2 complex accommodating the cationic endohedral metallofullerenes Li^+^@C_60_ as guest molecules, only the second reported example of a 1 : 2 host–guest complex involving Li^+^@C_60_ and the first within a CPP-based double nanohoop architecture. Evidently, the noncovalent attractive bonding exceeds the Coulomb repulsion of the two positive charges. These findings demonstrate that the intrinsic binding capability of the host is not fundamentally limited to 1 : 1 complexation but rather constrained by solid-state packing effects, suggesting that rational crystal engineering may enable access to such doubly occupied architectures in the condensed phase.

## Abstract 2

Mass spectrometry evidence of elusive 1 : 2 complexes of peropyrene-linked double nanohoop with C_60_, [6]CPP and Li^+^@C_60_ indicates that packing, not intrinsic binding, limits host occupancy in these nanohoops in the solid state.

## Graphical abstract

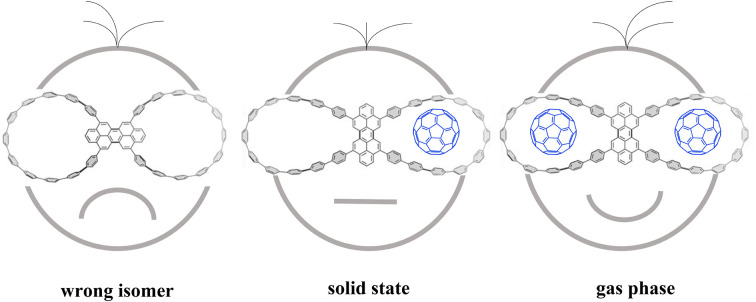

## Introduction

Cycloparaphenylenes ([*n*]CPPs) have attracted considerable research interest in recent times. The [*n*]CPP nanohoops are composed of “*n*” *para*-linked phenylene groups. Since their first successful synthesis by Bertozzi, Jasti and co-workers in 2008,^1^ there has been comprehensive coverage of their synthesis^2–5^ and modifications^5–9^ as well as properties^2,5,10–12^ and applications.^8,10,13^ Their curved cylindrical structure with its radially oriented π-orbitals yields a cavity that enables alteration of their properties through the variation of noncovalently linked molecular partners. This tunability underscores the need for a better understanding of the host–guest chemistry of the CPPs.^6,7,9,11,12^

**Fig. 1:**
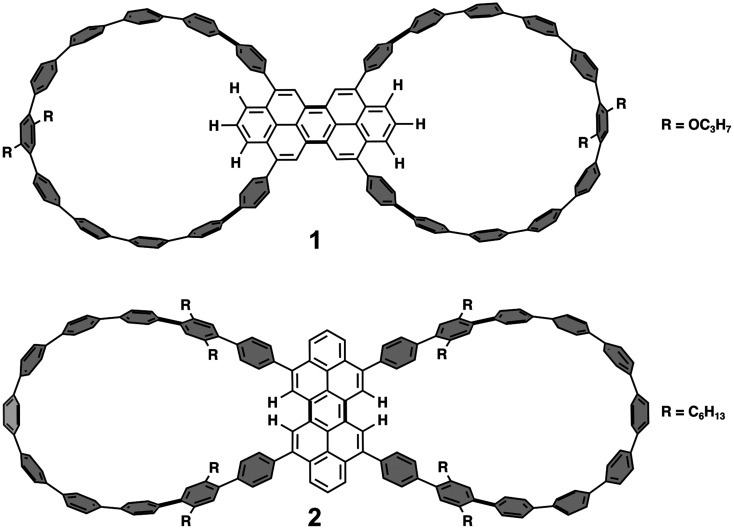
Structures of the double nanohoops 1 and 2 (CPP units shown in gray; peropyrene units shown in white).

The synthesis and study of double nanohoops represent an opportunity in which material properties can be controlled through the encapsulation of multiple guest molecules. The rapidly developing field of dimeric nanohoops has been reviewed recently.^6,9^ The common structural motif of dimeric CPPs consists of a central linker from which two CPP rings are extending. Obviously, the double nanohoop should operate as a host for two appropriate guest molecules, for which fullerenes and/or smaller CPP rings are particularly suitable choices. The double nanohoop 1 (Fig. 1) represents a recent representative of this new class of CPP dimers.^14^

Unfortunately, 1 is not suited to accommodate C_60_ and in an earlier study no complexation could be found. Steric hindrance is the most probable cause of this failure as three of the hydrogen atoms at the peropyrene termini are pointing into the CPP cavity, reducing its available space.^14^ As an alternative double nanohoop, compound 2 was synthesized, in which the CPP lassos extend from the bay region of the peropyrene.^15^ In this arrangement, the position of the corresponding hydrogen atoms is much less space demanding leading to a cavity that is more suitable for encapsulation. Moreover, the CPP rings possess an oval-shaped geometry. In fact, complexation of 2 with C_60_ was successfully observed. However, the desired 1 : 2 complex remained elusive and only the 1 : 1 complex could be obtained.^15^ The reason for this has to be seen in the unique solid-state packing of 2. Commonly, CPPs adopt a herringbone packing structure, but 2 features a unique lamellar packing motif which allows the CPP loops to form diagonal columns. Besides the larger intermolecular distance, the complex of 2 with C_60_ showed an almost identical packing structure to pristine 2 itself. This tight lamellar packing motif may have prevented the 1 : 2 complexation as this would have resulted in significant steric repulsion of neighbouring C_60_ molecules. However, provided the two cavities are matching the size of the guest molecule, double nanohoops are found to readily produce 1 : 2 host–guest complexes.^16–18^ Less predictable is the outcome if access to one of the cavities is sterically hindered like in the study by Fang *et al.* who observed the formation of 2 : 1 and 2 : 3 host–guest complexes of double nanohoops and C_60_.^19^ The failure of nanohoop 2 to produce 1 : 2 complexes was not only a surprise but also to some extend a disappointment as it was synthesized exactly for this purpose. The 1 : 1 and 1 : 2 complexes of 2 with C_60_ have been the subject of theoretical investigations (Density Functional Theory) into photoinduced electron transfer processes.^20^ It was found that charge transfer from the CPP groups to the C_60_ is energetically favoured, occurring on a sub-nanosecond time scale. Potential applications of such complexes in organic photovoltaics would be based on electron transfer as the key process in organic solar cells, involving the CPP ring as electron donor and C_60_ as electron acceptor. In order to be able to study this important aspect of the host molecule 2, the present investigation turns to electrospray ionization mass spectrometry (ESI-MS) which operates in the gas phase and/or at the border to the liquid phase. ESI enables the transfer of host–guest complexes from solution into the gas phase but can also promote their formation through aggregation processes during solvent evaporation within the ESI process.^21–23^ We have successfully employed ESI in a number of recent investigations into the host–guest chemistry of CPP-based systems.^24–33^

## Results and discussion

**Fig. 2:**
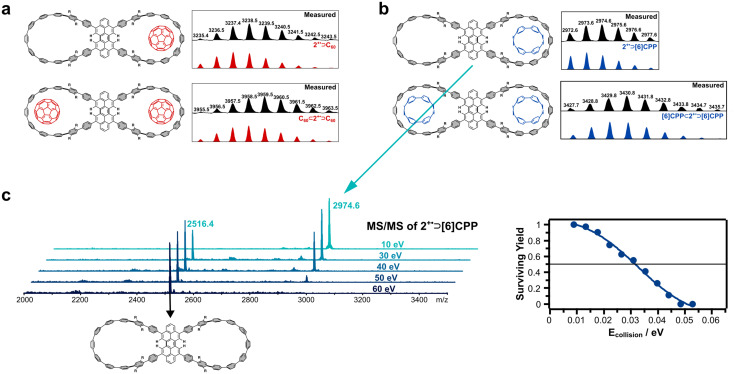
Isotope pattern of 2 with C_60_ (a) and with [6]CPP (b). MS^2^ experiment of 2˙^+^ ⊃ [6]CPP and its breakdown graph are displayed in c.

In a first set of experiments, we tested the ability of 2 to accommodate C_60_ and [6]CPP as guest molecules. C_60_ was chosen because of the reported failure of double occupation of the host molecule^15^ and [6]CPP was tested as an alternative guest molecule. In a recent comprehensive gas-phase study, the stability of ring-in-ring CPP complexes ([*n*]CPP ⊃ [*m*]CPP) was tested.^30^ It was found that the most stable complexes featured a size difference of 5 to 6 phenylene rings (*n* – *m* = 5 or 6). As 2 features cavities that lie in between [11]CPP and [12]CPP, [6]CPP represents a guest molecule with the ideal size match. Fig. 2a provides evidence of single and double attainment of C_60_ as the guest of the double nanohoop host molecule. Related complexes are observed for [6]CPP as the guest molecule (Fig. 2b). The 1 : 1 and 1 : 2 complexes of [6]CPP and 2 are the first binary ring-in-ring complexes involving a CPP-based double nanohoop. Earlier double nanohoop complexes featured exclusively fullerenes as the guest molecules. The assignment is based on the nominal *m*/*z* values in conjunction with the excellent match of measured and calculated isotope patterns. Fig. 2c displays the outcome of a MS^2^ experiment with the 1 : 1 ring-in-ring complex 2˙^+^ ⊃ [6]CPP. In this experiment, 2˙^+^ ⊃ [6]CPP is isolated and collisionally activated to promote its collision-induced dissociation (CID). The CID experiment reveals that [6]CPP is lost as the neutral and 2˙^+^, the radical cation of 2, is observed as the only fragment ion. It is interesting that the double nanohoop is thus the carrier of the positive charge in the complex. In our earlier investigations into CPP-in-CPP complexes it was found that always the inner CPP ring was ionized while the outer CPP host stayed neutral.^30,34^ We assume that the peropyrene linker in 2 is easier to oxidize than the [6]CPP moieties which would explain this observation. Both [6]CPP^35^ and aryl-substituted peropyrenes^36^ do possess low-lying oxidation potentials. However, the available literature data does not allow a more conclusive insight.

Although our experiments provide unequivocal evidence of the formation of 1 : 1 and 1 : 2 complexes of the double nanohoop with C_60_ and [6]CPP, the complex formation was accompanied by several other unwanted products also produced in the ESI process. This is evident from the ESI mass spectra shown in full in the SI (Fig. S1 and S2). Also, the careful variation of the experimental conditions did not improve the situation.

**Fig. 3:**
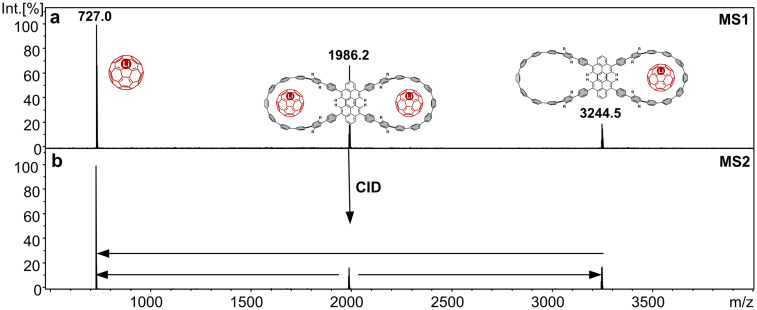
(a) Positive-ion ESI mass spectrum of a solution of DMF : Tol (1 : 1) containing 2 ⊃ Li^+^@C_60_ and (b) MS^2^ spectrum of Li^+^@C_60_ ⊂ 2 ⊃ @C_60_ recorded at a collision energy of 50 eV.

However, testing the cationic Li^+^@C_60_ endohedral metallofullerene as a guest gave a spectacular result, leading to a clean spectrum with the 1 : 1 and 1 : 2 complexes as the only observed products (Fig. 3a and Fig. S3). Li^+^@C_60_ is known to bind substantially stronger to [10]CPP than C_60_.^29,37,38^ This was found in early experiments by Itami and co-workers^37^ and confirmed by computational work of Sola and co-workers.^38^ Only recently it was possible to quantify these findings experimentally. ITC (Isothermal Titration Calorimetry) experiments by the Perez-Ojeda group revealed that the association constant for the Li^+^-containing complex^29^ was two orders of magnitude larger than that for the corresponding C_60_ complex. This is not only a consequence of the positive charge of Li^+^@C_60_ compared to neutral C_60_ as the guest, as Li^+^@C_60_ also binds stronger than the positively charged C_60_˙^+^ radical cation. While the positive charge of the cationic metallofullerene guest certainly increases the stability of the complex, given the negatively polarized CPP cavity which enhances attractive binding, the key effect that increases the complex stability with Li^+^@C_60_ is attributed to the uniform delocalization of the positive charge over the sphere. This homogeneity of the positive charge distribution enables a more uniform and therefore stronger interaction with the CPP ring.

Employing Li^+^@C_60_ as the guest, the formation of Li^+^@C_60_ ⊂ 2 ⊃ Li^+^@C_60_ is truly remarkable with double nanohoop 2 now accommodating two cations (Fig. 3a). Obviously, the noncovalent attractive forces exceed the Coulomb repulsion of the two positive charges. Note that although the 1 : 2 complex is heavier than the 1 : 1 complex its signal appears at lower *m*/*z* value as *z* = 2 for this doubly charged ion. The 1 : 2 complex is only the second stable dicationic complex in this context, following our previous report of the stable dicationic ([10]CPP ⊃ Li@C_60_)^2+^ complex.^29^ Given the fact that further electrochemical oxidation of Li^+^@C_60_ is hard, while CPP rings are relatively easy to oxidize, the ([10]CPP ⊃ Li@C_60_)^2+^ is most likely the noncovalent complex of a [10]CPP˙^+^ radical cation and Li^+^@C_60_ and thus also an example where the noncovalent attractive forces are stronger than the Coulomb repulsion.

A further observation from the ESI mass spectra of 2 (depicted in full in the SI, Fig. S1 and S2) refers to the efficient formation of the dimer ion of 2, *i.e.* (2)_2_^+^. The dimer formation most certainly results from the interaction of the two peropyrene moieties since we can exclude the formation of noncovalent dimers from the CPP moieties in the present experiments. There are also aggregates observed, including two molecules of 2 and C_60_ molecule(s). Unfortunately, the present instrumentation does not allow CID experiments beyond *m*/*z* 3000 that would have helped to elucidate possible ion structures.

The CID mass spectrum of Li^+^@C_60_ ⊂ 2 ⊃ Li^+^@C_60_ at 50 eV collision energy shows signals for only two fragment ions (Fig. 3b): one relatively weak signal corresponds to the 1 : 1 complex and a very abundant signal for Li^+^@C_60_. This pattern is in line with a scenario for the dissociation in which the 1 : 2 complex releases one Li^+^@C_60_. Such a reaction is known as Coulomb explosion in tandem mass spectrometry and refers to the dissociation of a doubly charged precursor ion into two singly charged fragment ions,^39,40^ and is often governed by the relief of Coulomb repulsion. This is followed by further dissociation of the 1 : 1 complex into Li^+^@C_60_ and the neutral double nanohoop. Li^+^@C_60_ is thus produced by both reactions and represents the final fragment ion, which explains its huge abundance in the MS^2^ mass spectrum. The 1 : 1 complex is only an intermediate in this dissociation sequence, hence its low abundance in the MS^2^ experiment. This is corroborated by the breakdown graph shown in the SI (Fig. S4), which displays the development of precursor and fragment ions as a function of the collision energy. Upon collision-induced decline of the 1 : 2 complex, Li^+^@C_60_ and the 1 : 1 complex are simultaneously formed and while the abundance of 1 : 1 complex eventually drops, Li^+^@C_60_ increases in abundance as the finally formed fragment ion.

**Fig. 4:**
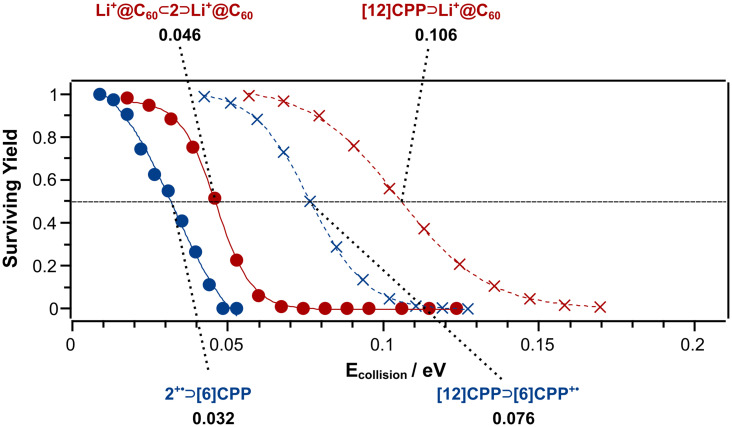
Breakdown graphs of the observed complexes of 2 compared with respective complexes featuring a [12]CPP host.

Finally, we assess the relative stabilities of the observed complexes. Notably, the doubly charged Li^+^@C_60_ ⊂ 2 ⊃ Li^+^@C_60_ (Fig. 4) is slightly more stable than the weakly bound ring-in-ring complex, 2˙^+^ ⊃ [6]CPP. However, compared with [12]CPP ⊃ Li^+^@C_60_ featuring a single CPP nanohoop as a host of comparable cavity size for which the energy-resolved decay is also depicted in Fig. 4, Li^+^@C_60_ ⊂ 2 ⊃ Li^+^@C_60_ is clearly less stable. The lower stability of Li^+^@C_60_ ⊂ 2 ⊃ Li^+^@C_60_ arises partly from the aforementioned Coulomb repulsion, which, however, cannot be quantified here. Moreover, there are less opportunities for π–π interactions provided by the cavity of the double nanohoop compared to the [12]CPP host. This is also evident from the comparison of 2˙^+^ ⊃ [6]CPP with [12]CPP ⊃ [6]CPP˙^+^. As a result of the enhanced possibilities for π–π interactions, the [12]CPP ⊃ [6]CPP˙^+^ complex is clearly more stable than 2˙^+^ ⊃ [6]CPP. This indicates that guest molecules like C_60_ and [6]CPP are less strongly attained in the double nanohoop compared to a [12]CPP host. Unfortunately, the instrumentation does not allow the study of source-generated precursor ions beyond *m*/*z* 3000 so that we cannot assess the stabilities of the complexes of 2 with C_60_. However, complexes with C_60_ can be expected to be more stable than those with [6]CPP and less than those with Li^+^@C_60_ as the guest.

## Conclusions

In summary, electrospray ionization operating at the boundary of liquid to gas phase provides evidence of the successful twin occupation of the double nanohoop host 2 which was prevented in the solid state. Next to the expected accommodation of C_60_ as the guest, we generated the first binary ring-in-ring nanohoop complex replacing both C_60_'s by much weakly bound [6]CPPs. Most remarkably, also cationic Li^+^@C_60_ was effectively incorporated twice into the double nanohoop with noncovalent binding surpassing Coulomb repulsion. Collision-induced dissociation experiments further corroborate the intrinsic stability of these supramolecular nanohoop assemblies and reveal their preferred charge localization, underscoring their potential for integration into photovoltaic devices.

These findings clearly demonstrate that double encapsulation of guests such as C_60_ or CPP is intrinsically feasible and was previously suppressed only by unfavorable—albeit rare—lamellar packing in the solid state. The results therefore motivate a renewed structural design of the double nanohoop scaffold: synthetically accessible modifications, for example, through strategic substitution to modulate intermolecular distances and packing motifs, could enable controlled double encapsulation and facilitate conductive wire formation in the solid state, as originally envisioned for these architectures.

## Conflicts of interest

The authors declare no conflict of interest.

## Supplementary Material

QO-013-D6QO00235H-s001

## Data Availability

All relevant data are within the article and its supplementary information (SI). Supplementary information is available. See DOI: https://doi.org/10.1039/d6qo00235h. The raw MS data underlying this study are openly available in the public repository Zenodo at https://zenodo.org/record/18745605 (https://doi.org/10.5281/zenodo.18745605).
