# Supplementary material for: Double encapsulation of C60, [6]CPP and Li+@C60 inside a peropyrene-linked, CPP-based double nanohoop
Source: Org Chem Front. 2026 Apr 8;13(11):3281–7. doi: 10.1039/d6qo00235h (PMC13089674; doi:10.1039/d6qo00235h)
Supplement: QO-013-D6QO00235H-s001 [file QO-013-D6QO00235H-s001.pdf]

Supplementary Information (SI) for Organic Chemistry Frontiers.
This journal is © the Partner Organisations 2026

Supplementary Information

Double encapsulation of C_{60}, [6]CPP and $Li^+@C_{60}$ inside a peropyrene-linked, CPP-based double nanohoop

Lei Ye[a], Yong Yang[b,c], Michal Juríček*[,b] and Thomas Drewello*[,a]

[a] Physical Chemistry I, Department of Chemistry and Pharmacy, Friedrich-Alexander-Universität Erlangen-Nürnberg, Erlangen, 91058 Germany

[b] Department of Chemistry, University of Zurich, Zurich, 8057 Switzerland

[c] School of Chemistry and Chemical Engineering, Southeast University, Nanjing, Jiangsu, 211189 China

* Email: michal.juricek@chem.uzh.ch, thomas.drewello@fau.de

Table of contents

1. Experimental section....S3

2. Additional measurements....S5

3. References....S9

1. Experimental section

Chemicals:

Double nanohoop **2** was synthesized following reported procedures[1]. [6]CPP was purchased TCI, $[Li^{+}@C_{60}](PF_6)^{-}$ from Idea International Co., Ltd, C_{60} and trifluoroacetic acid (TFA) from Merck. The solvents dichloromethane (DCM), dimethylformamide (DMF), *o*-dichlorobenzene (*o*-DCB) and toluene (Tol) were purchased from VWR (HPLC grade). All chemicals purchased were used without further purification.

Sample preparation:

Stock solutions of **2** were prepared in DCM (0.3 g l^{-1}), of $[Li^{+}@C_{60}](PF_6)^{-}$ in *o*-DCB (0.5 g l^{-1}), of C_{60} in Tol (0.4 g l^{-1}), and of [6]CPP in DCM (0.2 g l^{-1}). For ESI experiments, analyte solutions were prepared such that each analyte was present at a concentration of 1 x 10^{-5} mol l^{-1} in a DMF/Tol (1:1, v:v) mixture, to which a small amount of TFA was added.

Instrumentation:

Mass spectrometry experiments were conducted using a quadrupole time-of-flight mass spectrometer (micrOToF-Q II, Bruker Daltonics, Bremen) equipped with an electrospray ionization (ESI) source. The samples were directly infused into the ESI source with a syringe pump at a flow rate of 180 µl h^{-1}. The temperature of the nitrogen counter flow was set to 180 °C, and a capillary voltage of -4.5 kV was applied. For MS^2 experiments, N_2 was used as the collision gas, generated by a Parker LCMS64 nitrogen generator (99.999 % purity) and a flow rate of 0.2 l min^{-1}.

Breakdown graphs:

Breakdown curves were constructed by plotting the Survival Yield (SY) as a function of the collision energy. SY is defined as the ratio of the intensity of the intact precursor ions to the total ion intensity after activation:

$$\mathrm{SY} = \frac{I_{precursor}}{I_{precursor} + \sum I_{fragment}} \qquad (1)$$

where $I_{precursor}$ is the intensity of the undissociated precursor ion and $\sum I_{fragment}$ represents the summed intensity of all fragment ions.

To enable direct comparison between different complexes, the collision energy was normalized by dividing the laboratory-frame energy (E_{lab}) by the number of degrees of freedom (DoF) of

the hosting double nanohoop **2** and [12]CPP. This energy scale was chosen based on our previous results[2]. This normalization assumes that the encapsulated guest species (C_{60}, $Li^+@C_{60}$ and [6]CPP) are effectively shielded from direct collisions within the host cavity. Accounting for differences in DoF compensates for size-dependent differences of precursor ions in energy distribution upon collision, enabling their direct comparison. The number of DoF was calculated according to Equation 2:

$$DoF = (3n - 6) \quad (2)$$

where n corresponding to the number of atoms in the host molecule.

The SY curves were acquired under multiple collision conditions and fitted with a sigmoid Boltzmann function. The collision energy at which 50 % of the complex has dissociated into their fragment ions (E_{50}), is used as a relative measure of complex stability. It should be noted that the reported fragmentation energies are comparative rather than absolute values with respect to the intrinsic dissociation energy.

2. Additional measurements

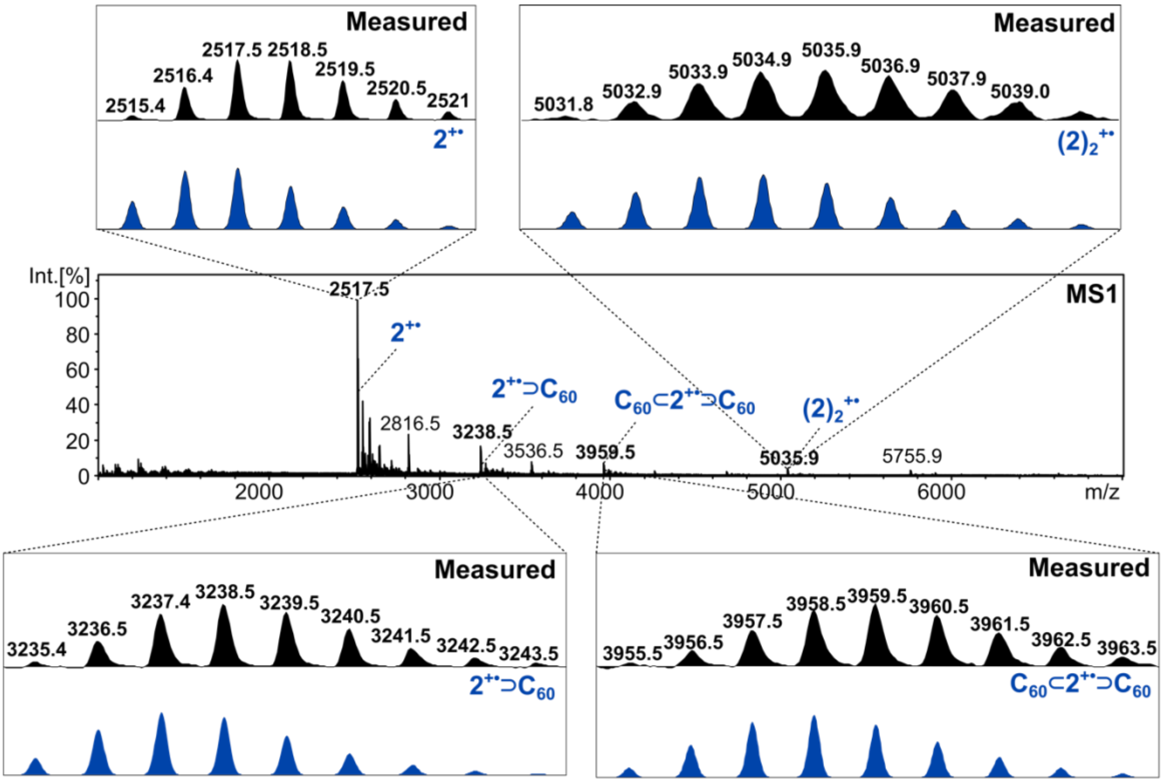

Figure S1: Positive-ion ESI mass spectrum (MS1) spraying a solution of DMF:Tol (1:1) containing double nanohoop **2** and C_{60}.

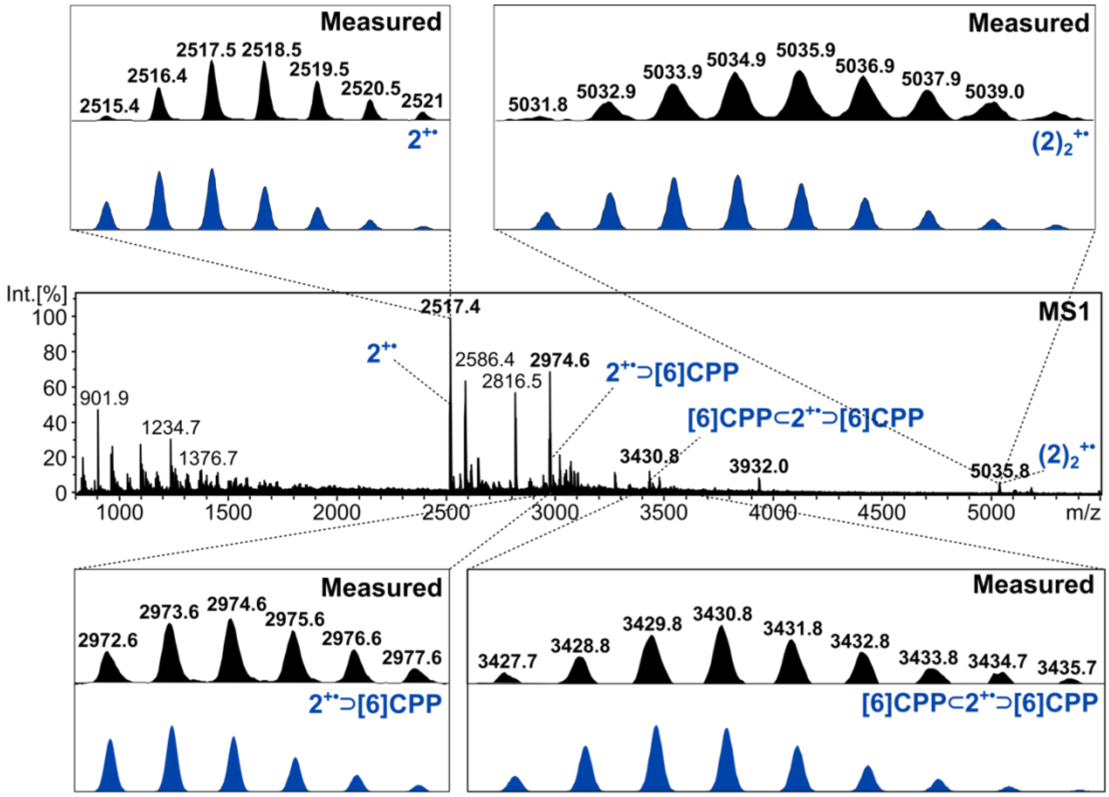

Figure S2. Positive-ion ESI mass spectrum (MS1) spraying a solution of DMF:Tol (1:1) containing double nanohoop **2** and [6]CPP.

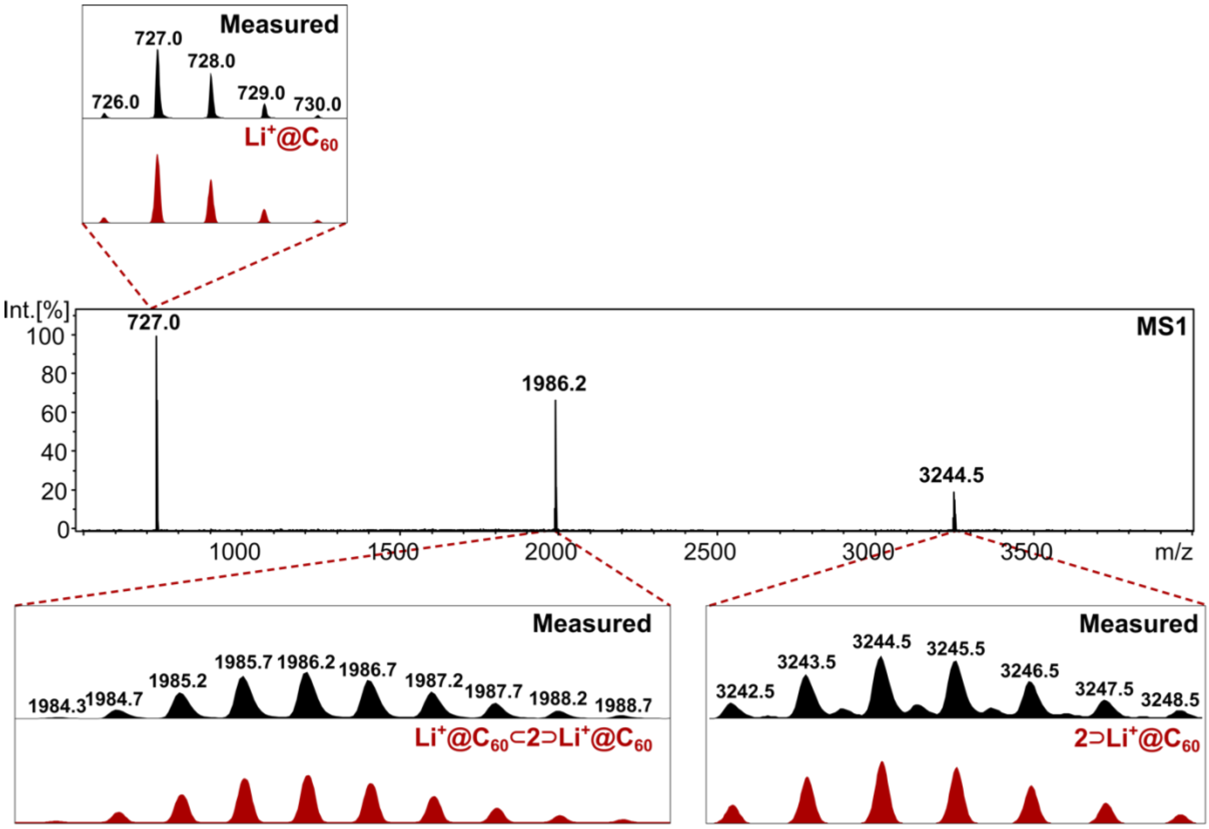

Figure S3. Positive-ion ESI mass spectrum (MS1) spraying a solution of DMF:Tol (1:1) containing double nanohoop **2** and $Li^{+}@C_{60}$.

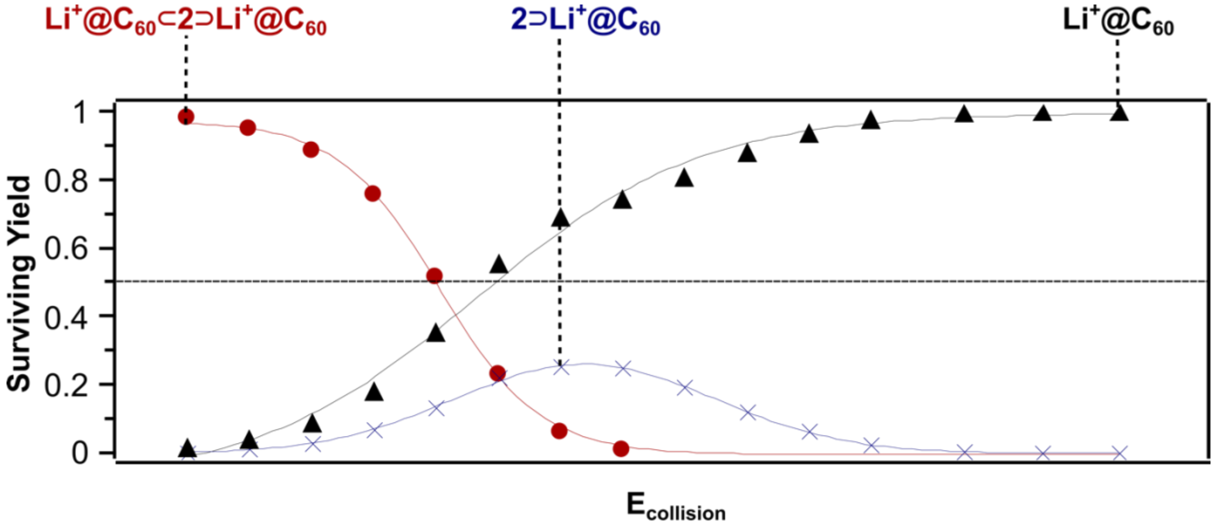

Figure S4: Breakdown graph (collision-induced dissociation) of $Li^+@C_{60}$⊂**2**⊃$Li^+@C_{60}$.

3. References

1. Y. Yang, S. Huangfu, S. Sato and M. Juríček, *Org. Lett.*, 2021, **23**, 7943–7948.
2. M. Freiberger, M. B. Minameyer, I. Solymosi, S. Fruhwald, M. Krug, Y. Xu, A. Hirsch, T. Clark, D. M. Guldi, M. von Delius, K. Amsharov, A. Gorling, M. E. Perez-Ojeda and T. Drewello, *Chemistry*, 2023, **29**, e202203734.
